# Clinical Patterns of Rocuronium and Cisatracurium Use in Acute Respiratory Distress Syndrome: A Retrospective Cohort Study

**DOI:** 10.3390/diseases14010022

**Published:** 2026-01-06

**Authors:** Imran Khan, Ariel Hendin, Bernadett Kovacs, Dominic Seguin, Caitlin Richler, Christine Landry, Pierre Thabet

**Affiliations:** 1 Montfort Hospital, Ottawa, ON K1K 0T2, Canada; arielhendin@montfort.on.ca (A.H.); bernadettkovacs@montfort.on.ca (B.K.); dominicseguin@montfort.on.ca (D.S.); caitlinrichler@montfort.on.ca (C.R.); christine.landry@uottawa.ca (C.L.); pierrethabet@montfort.on.ca (P.T.); 2 Faculty of Science, School of Pharmaceutical Sciences, University of Ottawa, Ottawa, ON K1H 8M5, Canada; 3 Institut du Savoir Montfort, Hôpital Montfort, Ottawa, ON K1K 0T2, Canada; 4 Department of Medicine, Faculty of Medicine, University of Ottawa, Ottawa, ON K1H 8M5, Canada; 5 Ottawa Hospital, Ottawa, ON K1Y 4E9, Canada

**Keywords:** acute respiratory distress syndrome, neuromuscular blockade, rocuronium, cisatracurium, ventilator dyssynchrony, critical care, mechanical ventilation, retrospective study, corticosteroids

## Abstract

Background: Neuromuscular blockade (NMB) is frequently used in moderate-to-severe acute respiratory distress syndrome (ARDS) to optimize ventilatory synchrony and minimize ventilator-induced lung injury. However, comparative real-world data on different NMB strategies remain limited. Objective: To describe patterns of neuromuscular blockade use in ARDS and describe clinical outcomes across four NMB strategies: intermittent rocuronium, continuous cisatracurium, escalation from rocuronium to cisatracurium, and de-escalation from cisatracurium to rocuronium. Methods: A retrospective chart review was conducted in an 18-bed tertiary ICU at Hôpital Montfort (Ottawa, Canada) between November 2021 and March 2025. Adult ARDS patients who received NMB for >24 h were included. Continuous variables (age, ventilation time, ICU stay) were summarized as means ± SD and median [IQR]; categorical variables (sex, ARDS etiology, mortality) as counts and percentages. Inferential testing was limited to baseline characteristics; clinical outcomes were summarized descriptively. Results: Fifty-one patients met inclusion criteria: rocuronium (*n* = 20), cisatracurium (*n* = 14), rocuronium→cisatracurium (*n* = 8), and cisatracurium→rocuronium (*n* = 9). Mean ventilation durations were 280, 195, 272, and 262 h, respectively; corresponding ICU stays were 245, 237, 380, and 299 h. Mortality ranged from 25% to 56%. Escalation from rocuronium to cisatracurium typically reflected persistent dyssynchrony or worsening oxygenation, whereas de-escalation occurred in improving patients with residual ventilatory drive. Variability in corticosteroid use, adjunctive proning, and epoprostenol were potential confounders. Conclusions: Distinct NMB use patterns in ARDS reflect bedside clinical judgment rather than predefined thresholds. Patient trajectory and dyssynchrony severity appear to drive NMBA escalation decisions more than oxygenation indices alone. These findings highlight the need for prospective studies defining standardized criteria for NMB initiation, escalation, and weaning in ARDS.

## 1. Introduction

Acute respiratory distress syndrome (ARDS) is a life-threatening condition characterized by severe hypoxemia, decreased lung compliance, and non-cardiogenic pulmonary edema. It remains a significant challenge in critical care medicine, with an estimated incidence of 10% of all ICU admissions and a mortality rate as high as 40% to 45% in severe cases [1]. The pathophysiology of ARDS involves diffuse alveolar damage, inflammatory infiltrates, and increased pulmonary vascular permeability, leading to impaired gas exchange and decreased lung compliance. Mechanical ventilation is a cornerstone of ARDS management, aimed at improving oxygenation and reducing the work of breathing. However, ventilatory support itself introduces several complications, including ventilator-induced lung injury (VILI) and patient self-inflicted lung injury (P-SILI) [2,3].

Ventilator dyssynchrony is common in mechanically ventilated patients with ARDS and reflects a mismatch between patient respiratory effort and ventilator-delivered support. Common forms include trigger, flow, cycle, and expiratory asynchrony, which may persist despite optimization of ventilator settings and sedation, particularly in the setting of severe hypoxemia and high respiratory drive [3,4]. In ARDS, elevated metabolic demand and increased work of breathing can overwhelm ventilatory adjustments, resulting in clinically significant dyssynchrony. Dyssynchrony has important clinical consequences, including increased work of breathing, respiratory muscle fatigue, elevated airway pressures, and lung over-distension, which may contribute to ventilator-induced lung injury and prolonged mechanical ventilation [4]. These effects often prompt the escalation of sedation and, in selected cases, the use of neuromuscular blockade to restore synchrony and protect the lung.

Neuromuscular blocking agents (NMBAs) are commonly used in patients with ARDS to improve ventilator synchrony and mitigate ventilator-induced lung injury (VILI) by suppressing spontaneous respiratory effort [5]. While NMBAs may reduce work of breathing and dyssynchrony, their use is associated with important risks, including prolonged neuromuscular weakness and ICU-acquired complications, necessitating careful selection of agent and dosing strategy [5,6].

Rocuronium and cisatracurium are among the most frequently used NMBAs in ARDS. Rocuronium, an aminosteroid NMBA with rapid onset and intermediate duration, is commonly administered as intermittent intravenous boluses, allowing flexible, targeted paralysis based on clinical need [7]. However, intermittent dosing may result in variable depth of paralysis and breakthrough dyssynchrony, with potential cumulative exposure following repeated boluses [8]. Cisatracurium, a benzylisoquinolinium NMBA, undergoes organ-independent Hofmann elimination and is typically administered as a continuous infusion, providing more consistent neuromuscular blockade and ventilator synchrony. However, continuous infusion requires careful titration to avoid excessive paralysis and prolonged neuromuscular weakness [5,6,7,8,9].

The optimal NMBA selection and dosing strategy in ARDS remain uncertain. The ROSE trial demonstrated no mortality benefit of early continuous cisatracurium infusion compared with lighter sedation strategies but suggested physiologic benefits in selected patients with severe ARDS [10]. Nonetheless, this trial was not designed to compare intermittent versus continuous NMBA strategies, and direct comparative data between rocuronium and cisatracurium are limited. Current guidelines support NMBA use in moderate-to-severe ARDS but do not define optimal agent selection or escalation strategies [4,5,11].

Both intermittent and continuous NMBA strategies have potential advantages and limitations [12]. Intermittent rocuronium boluses offer rapid onset and flexible, short-acting paralysis but may result in variable depth of neuromuscular blockade and breakthrough ventilator dyssynchrony. In contrast, continuous cisatracurium infusion provides more consistent paralysis and ventilator synchrony but may increase cumulative drug exposure, risk of ICU-acquired weakness, and overall cost [13,14].

This study examines real-world patterns of NMBA use in mechanically ventilated ARDS patients and describes clinical outcomes associated with four strategies: (A) rocuronium only, (B) cisatracurium only, (C) escalation from rocuronium to cisatracurium, and (D) de-escalation from cisatracurium to rocuronium. Outcomes of interest include duration of mechanical ventilation, documented ventilator dyssynchrony episodes, ICU length of stay, and hospital mortality.

## 2. Methods

This retrospective cohort study was conducted at Hôpital Montfort, a tertiary care academic hospital in Ottawa, Canada with an 18-bed level Intensive Care Unit. Adult patients (≥18 years) admitted to the intensive care unit (ICU) between 1 November 2021, and 31 March 2025, were screened for inclusion. Eligible patients met Berlin criteria for acute respiratory distress syndrome and received NMBA for a duration exceeding 24 h during invasive mechanical ventilation. Patients were identified through institutional pharmacy records and electronic medical charts using a combination of diagnosis codes for ARDS and pharmacy dispensing records for rocuronium and cisatracurium.

Patients were excluded if they received continuous renal replacement therapy (CRRT) during the NMBA treatment window, had documented contraindications to either rocuronium or cisatracurium, or had pre-existing neurological disorders (e.g., neuromuscular diseases, seizures) that could confound the assessment of ventilator synchrony or clinical outcomes. Additional exclusions included patients receiving concurrent therapies that could significantly interfere with NMBA pharmacodynamics or respiratory drive assessment (e.g., high-dose barbiturates or non-standard sedation protocols).

**Figure 1:**
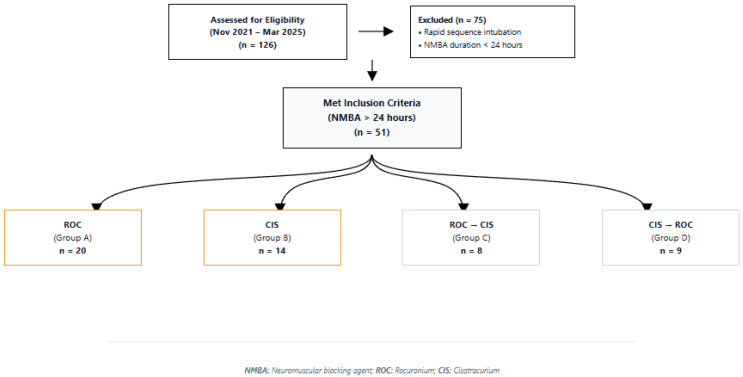
Study flow diagram.

During the study period (1 November 2021 to 31 March 2025), 126 adult ICU patients received neuromuscular blocking agents (NMBAs). Of those patients, 75 were excluded according to pre-specified exclusion criteria, namely NMBA use for rapid sequence intubation or for a duration of less than 24 h. The remaining 51 adult ICU patients met the inclusion criteria, having received neuromuscular blockade with either rocuronium or cisatracurium for more than 24 h. Of these, 20 patients were managed with intermittent rocuronium bolus therapy and 14 received continuous cisatracurium infusion. Sequential NMBA strategies were also observed: 8 patients initially treated with rocuronium were escalated to cisatracurium infusion, while 9 patients initially managed with cisatracurium were subsequently de-escalated to intermittent rocuronium. Patient screening, exclusions, and NMBA treatment allocation are summarized in Figure 1.

Eligible patients were stratified into four groups based on the NMBA regimen administered: (A) rocuronium only, (B) cisatracurium only, (C) rocuronium followed by cisatracurium, and (D) cisatracurium followed by rocuronium. The sequence of NMBA use was determined by clinician discretion and availability, without protocolized criteria. Dyssynchrony episodes were recorded when they required intervention (e.g., NMBA bolus, infusion titration, or sedation adjustment). For continuous infusion patients, dyssynchrony episodes were not consistently documented and therefore not extracted for this group. Ethics approval was obtained from the Montfort Hospital Research Ethics Board (Institut du Savoir Montfort, file number: 24-25-01-053).

## 3. Data Collection

The data were extracted from patient records using a standardized abstraction tool. Variables collected included demographics (age, sex, weight), initial and final partial pressure of oxygen in arterial blood (PaO_2_) to the fraction of inspired oxygen (FiO_2_) ratio, P/F ratios, corticosteroid exposure, sedation regimen, ventilator parameters, NMBA dosing characteristics (daily dose, cumulative exposure, duration), and episodes of ventilator dyssynchrony documented in clinical notes or respiratory therapy logs. Clinical outcomes assessed included total duration of mechanical ventilation, frequency of ventilator dyssynchrony, ICU length of stay, and hospital mortality.

## 4. Statistical Analysis

This study was designed as a descriptive, retrospective cohort study to characterize real-world patterns of neuromuscular blocking agent use in ARDS across four treatment strategies. Selection and sequencing of NMBAs were determined by the treating clinical team and were not protocolized. Given modest sample sizes, heterogeneity in trajectories, and no adjustment for illness severity, analyses were primarily descriptive and hypothesis-generating.

Baseline characteristics were compared across groups to assess for major imbalances. Continuous baseline variables were evaluated for normality using visual inspection and the Shapiro–Wilk test. To describe distributional characteristics in small samples, continuous variables are presented as the mean ± standard deviation (SD) and median with interquartile range [IQR]. For statistical comparisons, normally distributed variables were compared using one-way analysis of variance (ANOVA), whereas non-normally distributed variables were compared using the Kruskal–Wallis test. Categorical variables are summarized as counts or percentages and were compared using the χ^2^ test or Fisher’s exact test, as appropriate. For multi-category variables with sparse cell counts (e.g., ARDS etiology), Fisher’s exact test was used to generate a single omnibus *p*-value across the four groups.

Reported *p*-values represent omnibus, unadjusted comparisons across all four groups and were used solely to evaluate baseline comparability; no post hoc pairwise testing or adjustment for multiple comparisons was performed. No inferential statistical testing was conducted for clinical outcomes, which are presented descriptively. Analyses were performed using Microsoft Excel, and a two-sided *p*-value < 0.05 was considered statistically significant for baseline comparisons only.

## 5. Results

This retrospective health records review included 51 adult patients with moderate-to-severe ARDS requiring invasive mechanical ventilation who received neuromuscular blockade (NMBA) for more than 24 h. Patients were stratified into four NMBA strategy groups based on real-world prescribing patterns: rocuronium-only (Group A, *n* = 20), cisatracurium-only (Group B, *n* = 14), escalation from rocuronium to cisatracurium (Group C, *n* = 8), and de-escalation from cisatracurium to rocuronium (Group D, *n* = 9).

### 5.1. Baseline Characteristics and ARDS Severity

Baseline characteristics were broadly comparable in age, body weight, sex distribution, ARDS etiology, sedation, corticosteroid use and baseline oxygenation severity at the time of neuromuscular blockade initiation (Table 1). Overall, these findings support interpreting subsequent outcome patterns descriptively and in the context of illness trajectory and practice variation rather than predefined treatment allocation.

### 5.2. Corticosteroid Exposure

Corticosteroid use was common across all groups but was highest among patients requiring NMBA escalation (Table 2). Mean daily methylprednisolone-equivalent doses were 77 mg in the rocuronium-only group, 44 mg in the cisatracurium-only group, 125 mg in the rocuronium-to-cisatracurium group, and 46 mg in the cisatracurium-to-rocuronium group, consistent with greater disease severity and inflammatory burden in patients requiring escalation.

### 5.3. Oxygenation Trends

Improvements in oxygenation were observed across all NMBA strategies, although the magnitude and durability of change varied (Table 2). In the rocuronium-only group, mean P/F ratio improved from 108 at initiation to 195 at the end of therapy. The cisatracurium-only group demonstrated a more modest increase in mean P/F ratio from 133 to 174. In sequential regimens, oxygenation improved incrementally: patients escalated from rocuronium to cisatracurium showed stepwise improvement (111 → 155 → 170), whereas patients de-escalated from cisatracurium to rocuronium experienced an initial improvement (96 → 187) followed by a decline after transition (to 136). These trends highlight that changes in P/F ratio did not consistently parallel dyssynchrony burden or overall clinical trajectory.

Ventilator dyssynchrony burden differed meaningfully across NMBA strategies. Patients managed with rocuronium-only experienced fewer documented dyssynchrony episodes (mean 3.1) compared with those requiring sequential strategies, particularly patients escalated from rocuronium to cisatracurium (mean 6.6 episodes) and those de-escalated from cisatracurium to rocuronium (mean 7.3 episodes). These findings suggest that sequential NMBA strategies were often employed in patients with persistent or refractory dyssynchrony rather than as predefined treatment pathways.

### 5.4. Mechanical Ventilation Duration and ICU Length of Stay

**Figure 2:**
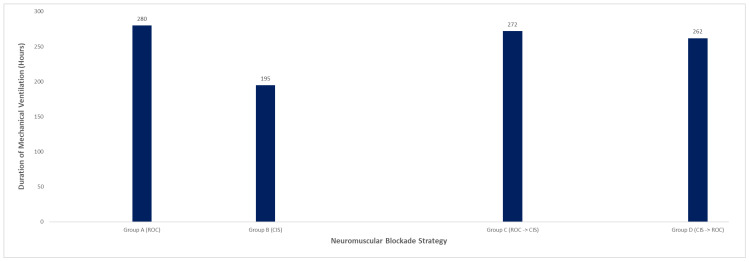
Ventilation Duration by NMBA Strategy of Rocuronium and Cisatracurium.

**Figure 3:**
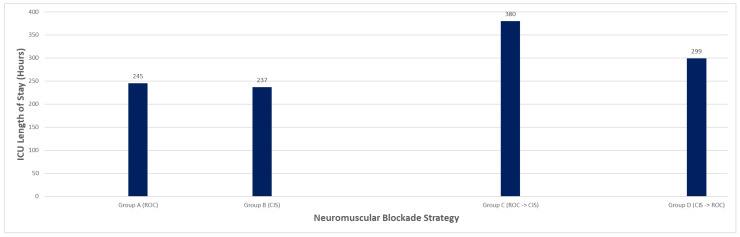
ICU Length of Stay by NMBA Strategy of Rocuronium and Cisatracurium.

Mechanical ventilation duration and ICU length of stay varied across strategies (Table 3; Figure 2 and Figure 3). Patients treated with rocuronium-only required a mean of 280 h of mechanical ventilation and had a mean ICU stay of 245 h. In contrast, patients managed with cisatracurium-only had shorter mean ventilation duration (195 h) and ICU stay (237 h). Sequential strategies were associated with longer ICU courses: patients escalated from rocuronium to cisatracurium had the longest mean ICU stay (380 h), while those transitioned from cisatracurium to rocuronium had a mean ICU stay of 299 h. These differences likely reflect illness severity and clinical trajectory rather than NMBA choice alone.

### 5.5. Mortality and Disposition

**Figure 4:**
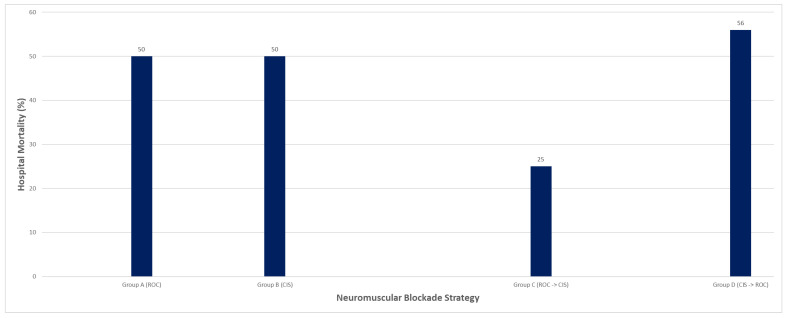
Hospital Mortality by NMBA Strategy of Rocuronium and Cisatracurium.

Hospital mortality ranged from 25% to 56% across groups (Table 3; Figure 4). Mortality was 50% in the rocuronium-only group, approximately 50% in the cisatracurium-only group (including three patients transferred for advanced support such as ECMO), 25% in the rocuronium-to-cisatracurium group, and highest in the cisatracurium-to-rocuronium group (56%).

Review of clinical records indicated that deaths were predominantly related to refractory hypoxemia, multi-organ failure, or underlying disease severity rather than complications directly attributable to NMBA strategy.

## 6. Discussion

Neuromuscular blockade remains an important adjunctive therapy in the management of moderate-to-severe ARDS, primarily to reduce ventilator–patient dyssynchrony, facilitate lung-protective ventilation, and mitigate ventilator-induced lung injury. Early evidence supporting continuous neuromuscular blockade in ARDS was largely derived from the ACURASYS trial, which demonstrated improved oxygenation, reduced barotrauma, and a modest mortality benefit with early cisatracurium infusion in patients with severe ARDS [15]. These findings contributed to widespread adoption of continuous cisatracurium infusion in select patients with refractory hypoxemia or severe dyssynchrony.

Subsequently, the ROSE trial challenged the routine use of early neuromuscular blockade by demonstrating no significant improvement in mortality or ventilator-free days when early continuous cisatracurium infusion was compared with usual care incorporating lighter sedation and selective NMBA use [10]. Importantly, the trial was designed to evaluate early routine neuromuscular blockade versus no routine paralysis, not to compare different NMBA agents, intermittent bolus dosing versus continuous infusion, or escalation strategies. Patients enrolled in the ROSE trial were severely ill, frequently required high PEEP, and received aggressive ventilatory support, which may have attenuated any incremental benefit of routine paralysis. As such, while ROSE informs decisions regarding when to initiate NMBA therapy, it provides limited guidance on which agent to use, how to dose, or when to escalate or de-escalate paralysis once NMBA therapy is deemed necessary.

In contemporary ICU practice, NMBA use is often individualized and reactive rather than protocolized. Decisions are influenced by patient trajectory, burden of dyssynchrony, ventilatory mechanics, and response to adjunctive therapies such as sedation optimization, proning, or inhaled pulmonary vasodilators. Our study was therefore not designed as a comparative efficacy trial but rather as a descriptive retrospective cohort aimed at characterizing real-world prescribing patterns surrounding neuromuscular blockade in a heterogeneous ARDS population.

Rocuronium bolus dosing plays a pragmatic and underexplored role in ARDS management beyond its traditional use for intubation. Its rapid onset, intermediate duration of action, and intermittent administration allow clinicians to target discrete episodes of ventilator dyssynchrony, agitation, or excessive ventilatory drive without committing patients to prolonged deep paralysis. This strategy may be particularly useful in patients with fluctuating respiratory mechanics, evolving sedation requirements, or early signs of clinical improvement. In our cohort, patients managed with rocuronium alone required longer durations of mechanical ventilation and ICU stay but experienced meaningful improvements in oxygenation. These findings likely reflect greater baseline illness severity and prolonged disease course rather than inferiority of the agent itself. While bolus dosing limits cumulative NMBA exposure and may reduce the risk of prolonged neuromuscular weakness, it may also permit breakthrough dyssynchrony between doses, prompting escalation in select patients.

Cisatracurium, administered as a continuous infusion, offers predictable pharmacokinetics via Hofmann elimination and sustained paralysis independent of hepatic or renal function. In our cohort, patients treated exclusively with cisatracurium had shorter ventilation durations and ICU stays. However, without statistical adjustment or formal baseline comparability analysis, these differences should not be interpreted as causative. Selection bias is likely, as cisatracurium may have been preferentially chosen in patients perceived to require consistent ventilator control or in those with evolving multi-organ dysfunction. Continuous infusion offers stable synchrony but requires infusion pump management, ongoing titration, and carries a potential risk of prolonged deep paralysis.

Sequential NMBA strategies reflect dynamic bedside decision-making rather than predefined escalation algorithms. Patients escalated from rocuronium to cisatracurium (ROC → CIS) typically exhibited persistent dyssynchrony, worsening oxygenation, or sustained high ventilatory drive despite intermittent bolus therapy. These patients experienced longer ICU stays, higher dyssynchrony burden, and modest incremental improvements in oxygenation, suggesting greater illness severity rather than delayed escalation alone. Conversely, patients transitioned from cisatracurium to rocuronium (CIS → ROC) were generally improving clinically but continued to experience residual dyssynchrony requiring intermittent control. The observed decline in P/F ratios following de-escalation underscores that oxygenation alone is an imperfect surrogate for ventilator synchrony and highlights the importance of clinical context.

Our detailed group-level findings further support these interpretations. Patients in the rocuronium-only group required a mean of 280 h of mechanical ventilation, experienced an average of 3.1 documented dyssynchrony episodes, and had a hospital mortality rate of 50%. Although oxygenation improved (P/F 108 → 195), no causal inference can be made. Patients in the cisatracurium-only group had shorter ventilation durations (195 h) and ICU stays (237 h), though baseline differences likely influenced these outcomes. Sequential strategies demonstrated the highest dyssynchrony burden and longest ICU stays, particularly in the ROC → CIS group, reinforcing that escalation typically occurred in patients with more severe or refractory disease.

Collectively, these findings emphasize that NMBA choice or sequencing alone does not determine patient-centered outcomes such as ventilation duration, ICU length of stay, or mortality. Instead, illness trajectory, residual dyssynchrony, and response to adjunctive ICU interventions appear more influential. Importantly, although the ROSE trial informs the broader question of routine early paralysis, it does not address the nuanced bedside decisions clinicians face once NMBA therapy is initiated—namely, agent selection, dosing strategy, and escalation thresholds. Our findings therefore highlight an important evidence gap and support the need for prospective studies directly comparing NMBA agents and dosing strategies within clinically relevant ARDS subpopulations.

Cost considerations may also influence neuromuscular blocking agent selection in ARDS. Using institutional acquisition pricing in Canadian dollars as an illustrative example, a 70 kg patient receiving cisatracurium at approximately 3 mcg/kg/min would incur an estimated drug acquisition cost of roughly CAD $250 per day (based on CAD $11.35 per vial), exclusive of additional resource utilization such as infusion preparation, nursing workload, neuromuscular monitoring, and supportive care measures (e.g., ophthalmic lubrication) [16]. In contrast, intermittent rocuronium bolus administration (50 mg per dose at approximately CAD $2.25 per vial, assuming an average of three doses daily) corresponds to an estimated acquisition cost of approximately CAD $6.75 per day [16]. Although cost was not a predefined outcome of this study, this marked difference highlights a pragmatic consideration in NMBA selection, particularly for patients who do not require continuous deep paralysis.

Several limitations should be acknowledged. First, the retrospective design relied on chart review and was subject to incomplete data capture and documentation bias. Providers without a standardized institutional scoring system identified ventilator dyssynchrony clinically, and documentation practices varied, which may have led to under-estimation of dyssynchrony events. NMBA selection and sequencing were based on provider discretion, and user-dependent factors such as sedation titration or delayed ventilator adjustments may have contributed to apparent dyssynchrony independent of ARDS pathophysiology. Detailed ventilator parameters—including PEEP, driving pressure, and tidal volume—were not consistently documented at the time of dyssynchrony events and therefore could not be analyzed. Similarly, adjunctive therapies known to influence oxygenation, such as prone positioning and inhaled prostaglandin agents (e.g., epoprosterenol), as well as cumulative NMBA exposure in sequential regimens, were not uniformly captured. Corticosteroid exposure varied across groups and may have influenced respiratory mechanics and outcomes. The cohort also included heterogeneous ARDS etiologies, including a small number of patients with status asthmaticus, whose additional bronchoconstrictive physiology may have affected ventilatory response to neuromuscular blockade. Finally, the modest sample size and single-center design limit generalizability and preclude causal inference, highlighting the need for prospective, standardized studies to better define optimal NMBA strategies and assessment of ventilator synchrony in ARDS.

In summary, this study provides real-world insight into the use of intermittent rocuronium boluses, continuous cisatracurium infusion, and sequential NMBA strategies in ARDS. While all approaches can achieve adequate ventilator control, escalation decisions appear driven by persistent dyssynchrony and clinical deterioration rather than oxygenation thresholds alone. Our findings reinforce the individualized nature of NMBA use in ARDS and underscore the need for future prospective studies comparing agents and dosing strategies to better guide bedside decision-making in this complex and heterogeneous population.

## 7. Conclusions

This retrospective cohort study describes real-world neuromuscular blockade strategies used in patients with moderate-to-severe ARDS requiring mechanical ventilation. Intermittent rocuronium boluses provided flexible, targeted control of ventilator dyssynchrony, whereas continuous cisatracurium infusion offered more predictable and sustained paralysis, particularly in patients with greater illness severity. Sequential escalation from rocuronium to cisatracurium was associated with higher dyssynchrony burden and longer ICU stays, reflecting worsening clinical trajectories rather than predefined thresholds. Overall, these findings suggest that persistent ventilator dyssynchrony and evolving patient physiology—rather than P/F ratios alone—primarily guide NMBA selection and escalation in clinical practice. Prospective studies are needed to define standardized escalation criteria and optimize NMBA use in heterogeneous ARDS populations.

## Figures and Tables

**Table 1:** Baseline Characteristics across Neuromuscular Blocking Agent Strategy Groups.

Characteristic	ROC (*n* = 20)	CIS (*n* = 14)	ROC → CIS (*n* = 8)	CIS → ROC (*n* = 9)	*p*-Value
Age (years)	60.9 ± 12.4 61 [52–70]	55.8 ± 11.1 56 [48–64]	46 ± 9.8 46 [39–53]	46.7 ± 10.2 47 [39–54]	0.08
Weight (Kg)	90.8 ± 18.6 90 [78–103]	83.3 ± 15.9 82 [72–94]	101.5 ± 21.4 100 [86–116]	90 ± 17.3 89 [77–102]	0.29
Sex Distribution (M/F)	11 M/9 F	8 M/6 F	5 M/3 F	6 M/3 F	0.89
ARDS Etiology (no. of cases)					0.27 ^†^
Severe Pneumonia—CAP	5	7	6	6	-
Severe Pneumonia—Aspiration	3	3	1	1	-
COVID Pneumonia	7	3	1	1	-
Status Asthmaticus	2	0	0	1	-
Sepsis—Unknown Source	1	1	0	0	-
Sepsis—Multi-organ failure	1	0	0	0	-
Sepsis—Abdominal Infection	1	0	0	0	-
Sedatives Used (% of patients)					
Midazolam	100%	71%	100%	100%	0.06
Propofol	50%	100%	100%	100%	0.01
Hydromorphone	75%	71%	100%	100%	0.18
Ketamine	10%	14%	38%	44%	0.12

Abbreviations: ROC—Rocuronium; CIS—Cisatracurium; ARDS: Acute Respiratory Distress Syndrome; CAP: Community Acquired Pneumonia; M/F—Male/Female PaO_2_/FiO_2_—Partial pressure of oxygen in arterial blood (PaO_2_) to the fraction of inspired oxygen (FiO_2_). ^†^ Global *p*-value derived from Fisher’s exact test comparing the overall distribution of ARDS etiologies across the four NMBA strategy groups. *p*-values are omnibus (global) comparisons across four groups, unadjusted, and intended for baseline comparability only; no post hoc pairwise testing was performed.

**Table 2:** Corticosteroid and Oxygenation requirements across Neuromuscular Blocking Agent Strategy Groups.

Characteristic	ROC (*n* = 20)	CIS (*n* = 14)	ROC → CIS (*n* = 8)	CIS → ROC (*n* = 9)	*p*-Value
Daily Corticosteroid dose (Methylprednisolone equivalent mg)	77 ± 42 70 [45–95]	44 ± 31 40 [25–60]	125 ± 58 120 [85–160]	46 ± 29 40 [28–55]	0.01
Baseline PaO_2_/FiO_2_ (P/F)	108 ± 34 104 [85–125]	133 ± 41 128 [105–155]	111 ± 29 110 [92–130]	96 ± 27 95 [80–110]	0.17

Abbreviations: ROC—Rocuronium; CIS—Cisatracurioum; M/F—Male/Female. PaO_2_/FiO_2_—Partial pressure of oxygen in arterial blood (PaO_2_) to the fraction of inspired oxygen (FiO_2_). *p*-values are omnibus (global) comparisons across four groups, unadjusted, and intended for baseline comparability only; no post hoc pairwise testing was performed.

**Table 3:** Outcomes by Neuromuscular Blocking Agent Strategies.

Group	Ventilation Time (hours)	ICU Stay (hours)	Hospital Mortality (%)
Rocuronium	280	245	50
Cisatracurium	195	237	50
Rocuronium → Cisatracurium	272	380	25
Cisatracurium → Rocuronium	262	299	56

## Data Availability

The datasets generated and/or analyzed during the current study are available from the corresponding author upon reasonable request and in accordance with institutional data-sharing policies.
